# Flexible wide-range multidimensional force sensors inspired by bones embedded in muscle

**DOI:** 10.1038/s41378-024-00711-7

**Published:** 2024-05-22

**Authors:** Jie Zhang, Xiaojuan Hou, Shuo Qian, Jiabing Huo, Mengjiao Yuan, Zhigang Duan, Xiaoguang Song, Hui Wu, Shuzheng Shi, Wenping Geng, Jiliang Mu, Jian He, Xiujian Chou

**Affiliations:** 1 Science and Technology on Electronic Test and Measurement Laboratory, North University of China, Taiyuan, 030051 China; 2 School of Software, North University of China, Taiyuan, 030051 China; 3 School of Mechanical Engineering, Hebei University of Architecture, Zhangjiakou, 075000 China; 4 HBIS Group Co. Ltd., Shijiazhuang, 050023 China

**Keywords:** Electrical and electronic engineering, Electronic devices, Structural properties

## Abstract

Flexible sensors have been widely studied for use in motion monitoring, human‒machine interactions (HMIs), personalized medicine, and soft intelligent robots. However, their practical application is limited by their low output performance, narrow measuring range, and unidirectional force detection. Here, to achieve flexibility and high performance simultaneously, we developed a flexible wide-range multidimensional force sensor (FWMFS) similar to bones embedded in muscle structures. The adjustable magnetic field endows the FWMFS with multidimensional perception for detecting forces in different directions. The multilayer stacked coils significantly improved the output from the μV to the mV level while ensuring FWMFS miniaturization. The optimized FWMFS exhibited a high voltage sensitivity of 0.227 mV/N (0.5–8.4 N) and 0.047 mV/N (8.4–60 N) in response to normal forces ranging from 0.5 N to 60 N and could detect lateral forces ranging from 0.2–1.1 N and voltage sensitivities of 1.039 mV/N (0.2–0.5 N) and 0.194 mV/N (0.5–1.1 N). In terms of normal force measurements, the FWMFS can monitor finger pressure and sliding trajectories in response to finger taps, as well as measure plantar pressure for assessing human movement. The plantar pressure signals of five human movements collected by the FWMFS were analyzed using the k-nearest neighbors classification algorithm, which achieved a recognition accuracy of 92%. Additionally, an artificial intelligence biometric authentication system is being developed that classifies and recognizes user passwords. Based on the lateral force measurement ability of the FWMFS, the direction of ball movement can be distinguished, and communication systems such as Morse Code can be expanded. This research has significant potential in intelligent sensing and personalized spatial recognition.

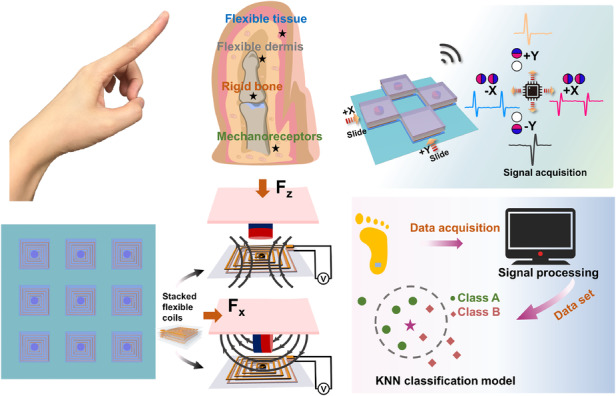

## Introduction

In recent years, with the development of flexible electronics, flexible pressure sensors have shown great potential in motion monitoring^1–3^, human‒machine interaction (HMI)^4–6^, personalized medicine^7–9^, and soft intelligent robots^10–12^. Perceiving and detecting multidirectional mechanical stimuli is crucial for pressure sensing and can provide comprehensive, complete, and accurate information about pressure distributions and interactions to realize motion direction detection^13^, slip detection^14^, and grasp detection^15^. Numerous studies have been conducted to achieve multidirectional force response and a wide sensing range in pressure sensors for various applications.

Depending on the sensitive mechanism, most flexible pressure sensors are based on piezoresistive^16–18^, piezoelectric^19–21^, capacitive^22–24^, electromagnetic^25–27^ or triboelectric effects^28–30^, which rely on reliable material selection, well-crafted sensor structural design and advanced manufacturing to achieve wide sensing ranges and miniaturization. Simultaneously, arrays are frequently employed as sensing units in the quest for detecting multidimensional forces, leading to inherent challenges such as multiple electrodes and complex wiring connection problems, in addition to the potential issue of signal crosstalk. The use of piezoelectric, electromagnetic, and triboelectric sensors as self-powered active sensors has become a trending research topic. They do not require an external power supply, greatly reducing the space volume. However, piezoelectric sensors are susceptible to depolarization, and triboelectric sensors are highly affected by the environment; thus, their applications are limited.

Electromagnetic flexible pressure sensors have attracted attention due to their stability, resistance to environmental effects, and magnetic field directionality. Soft magnetic materials with a predesigned magnetic distribution are often used to precisely locate the position of the force. For example, Yan et al.^31^ proposed a soft tactile sensor with self-decoupling and super-resolution abilities by designing a sinusoidally magnetized flexible film for robotic manipulation. The sensor can measure both normal force and lateral force and has a pressure sensing range of only 8 N. Man et al.^32^ proposed a tactile and airflow motion sensor based on flexible double-layer magnetic cilia. The upper layer of the magnetic cilia is a flexible material mixed with magnetic particles, while the lower layer is a pure flexible material. The sensor has a detection range between 0 and 60 µN. In addition, Zhou et al.^33^ presented a multifunctional and self-powered electronic skin system based on a whisker-like magnetized microcilia array. The electronic skin has a global area of ≈3.15 cm^2^ and exhibits a high sensitivity of 0.04 mV kPa^−1^ (0.03–5 kPa) and 0.012 mV kPa^−1^ (5–26 kPa) with a wide detection range of 35 kPa. In summary, most magnet-based flexible pressure sensors incorporate magnetic particles into flexible substrates to provide a magnetic field for the sensors. They are strongly affected by the geomagnetic field and have a low output performance and a narrow working range. This can be attributed to the limited quantity of magnetic particles, resulting in a relatively low level of magnetization. Furthermore, the elastic substrate absorbs part of the mechanical energy, thereby diminishing the magnetic field strength within the coil.

Inspired by the finger structure of humans, which consists of bones embedded in muscles and skin tissue wrapped around the periphery, this paper proposes a flexible wide-range multidimensional force sensor (FWMFS). The FWMFS is based on a rigid magnet with an adjustable magnetic field and multilayer stacked coils, which can efficiently enhance the performance of flexible sensors. The optimal sizes of the rigid part and the flexible coil are obtained through numerical analysis. The FWMFS can achieve a normal force measurement range of 0.5–60 N and distinguish signals from different stimulus directions based on the arrangement of the magnetic field. The FWMFS exhibits high voltage sensitivities of 0.227 mV/N (0.5–8.4 N) and 0.047 mV/N (8.4–60 N) in response to normal forces. The FWMFS can detect lateral forces ranging from 0.2–1.1 N with voltage sensitivities of 1.039 mV/N (0.2–0.5 N) and 0.194 mV/N (0.5–1.1 N). The FWMFS has various applications for single normal force measurement and multidimensional force measurement. When the FWMFS is integrated into the sole of a shoe, the plantar pressure signals are extracted, and the subspace k-nearest neighbors (k-NN) algorithm is employed to process the sensor information. This allows for the classification and recognition of different human motion states, achieving a recognition accuracy of 92%. The FWMFS can be employed to monitor not only different finger pressures but also sliding trajectories through the array. Moreover, an artificial intelligence biometric authentication system is being developed that classifies and recognizes user passwords according to user habits, improving password security and providing a new approach to digital network security. Depending on the sensitivity of the sensor to stimuli in different directions, it becomes possible to distinguish the direction of ball movement and optimize communication systems such as Morse Code. The directional differentiation of the FWMFS is expanded from two-dimensional to four-dimensional by the array, offering promising applications in human–computer interaction and wearable intelligent sensing.

## Results and discussion

### Concept and design of the FWMFS

**Fig. 1:**
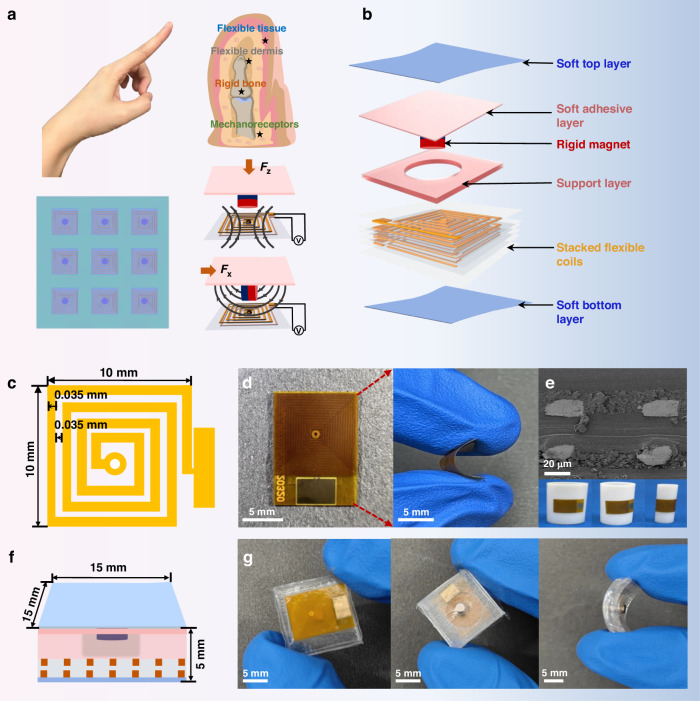
Concepts and structure of the FWMFS. **a** Diagram of the human finger, FWMFS and FWMFS arrays. **b** Structure of the FWMFS. Size (**c**), photographs (**d**) and SEM images (**e**) of the flexible MEMS coils. Size (**f**) and photographs (**g**) of the FWMFS

Inspired by the structure of the human finger (Fig. 1a), a flexible wide-range multidimensional force sensor (FWMFS) with flexible multilayer stacked MEMS coils is proposed. Adjustment of the magnetic field arrangement allows the FWMFS to distinguish the direction of the stimulus. As shown in Fig. 1b, the FWMFS is composed of a soft top layer, a soft adhesive layer, a rigid magnet, a support layer, a stacked flexible coil, and a soft bottom layer. The core components of the FWMFS are a rigid magnet (1.25 mm in radius and 4 mm in height) and flexible coils, which mimic the internal bone and surrounding muscle tissue of the human finger. The soft adhesive layer (2 mm thick) and the support layer (2 mm thick) transmit the force to the rigid magnet, causing the magnet to move. The sensing layer, consisting of flexible multilayer coils, converts the external stimulus into an electrical signal by electromagnetic induction. The soft top layer and the soft bottom layer mimic the skin of the human finger and are as thin as 0.016 mm, providing protection for the internal structure. The flexible coils have a line width and a line pitch of 0.035 mm; the entire size of the multilayer coils is 13 × 10 × 0.5 mm^3^, and the coils can be attached to different spatial surfaces, as shown in Fig. 1c, d. The SEM image is shown in Fig. 1e. In Fig. 1f, the FWMFS has an overall dimension of 15 × 15 × 5 mm^3^. Figure 1g shows an optical image of the FWMFS.

### Working principle and simulation analysis

**Fig. 2:**
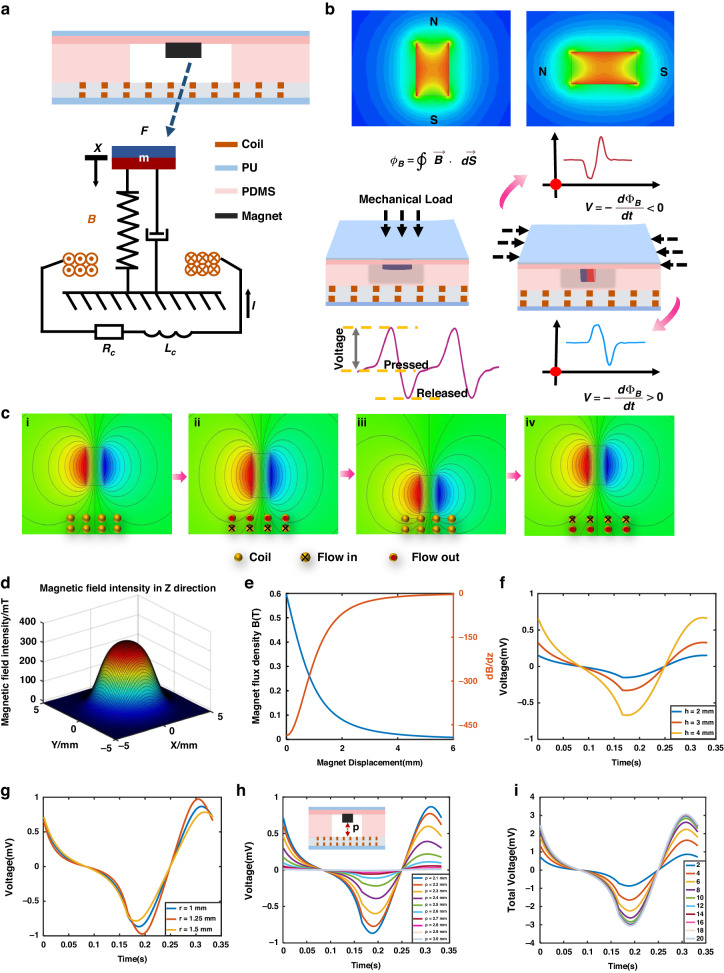
Working principle and simulation analysis. **a** Theoretical model. **b** Mechanism of FWMFS multidimensional sensing. **c** Working principle. **d** 3D image of the magnetic field intensity. **e** Relationships between the magnet flux density, magnetic field change rate, and magnet displacement. **f** Relationship between the output voltage of the FWMFS and the magnet height. **g** Relationship between the output voltage of the FWMFS and the magnet radius. **h** Output voltage when flexible MEMS coils are used at different positions. **i** Relationship between the FWMFS total output voltage and flexible coil layers

As shown in Fig. 2a, the FWMFS is a laminated structure consisting of a soft top layer, a soft adhesive layer, a rigid magnet, a support layer, stacked flexible coils, and a soft bottom layer. The soft adhesive layer, rigid magnet, and support layer form the core module of the electromagnetic pressure sensor, which can be equated to a second-order spring-damper-mass system. The force balance equation of the system given by Newton’s second law can be represented by Eq. (1).

1 $$m\ddot{z}+c\dot{z}+kz=F$$

where *m* is the magnet mass, *z* is the magnet displacement, *c* is the damping coefficient, *k* is the equivalent spring factor, and *F* is the external force.

The above equation can also be written as:

2 $$m\frac{{d}^{2}z}{d{t}^{2}}+c\frac{dz}{dt}+k{\rm{z}}=m\frac{{d}^{2}y}{d{t}^{2}}$$

where *y* is the displacement of external motivational forces, and the undamped intrinsic angular frequency of the system is ${\omega }_{n}=\sqrt{\frac{k}{m}}$.

The damping ratio is $\zeta =\frac{c}{2\sqrt{km}}$.

The gain (Gain) and phase shift ($\varphi$) of the sensor steady-state displacement can be expressed by Eq. (3) and Eq. (4).

3 $$Gain(j\omega)=\frac{-1}{1-{(\omega /\omega_{0})}^{2}+2\xi j(\omega /\omega_{0})}$$

4 $$\varphi (\omega )=\arctan \left[\frac{2\xi }{(\omega /{\omega }_{0})-({\omega }_{0}-\omega )}\right]$$

where $\omega$ is the input frequency of the system.

This second-order system is packaged as a complete sensor system through the top and bottom layers, and the packaged system can be equated to an additional spring-damper-mass system, so the whole system is connected in series as a two-degree-of-freedom vibration system. This approach will introduce new gains to the system, and to ensure the output linearity of the sensor-mechanical system, the system must have good dynamic characteristics.

Assuming that the displacement of the topmost layer of the sensor is $y={y}_{0}\,\sin \omega t$, and therefore, the input acceleration is denoted as $a=-{y}_{0}{\omega }^{2}\,\sin \omega t$, the equations of motion for the tandem system are as follows:

5 $$\left\{\begin{array}{l}{m}_{1}{x}_{1}^{\text{'}\text{'}}+{c}_{2}{x}_{1}^{\text{'}}+{k}_{1}{x}_{1}-{k}_{2}({x}_{2}-{x}_{1})={k}_{1}{y}_{0}\,\sin wt+{c}_{1}w{y}_{0}\,\cos wt\\ {m}_{1}{({x}_{2}-{x}_{1})}^{\text{'}\text{'}}+{k}_{1}({x}_{2}-{x}_{1})=-{m}_{2}{({x}_{2}-{x}_{1})}^{\text{'}\text{'}}\end{array}\right.$$

where *k*_*1*_ and *k*_*2*_ are the equivalent stiffnesses of the encapsulated body and the sensitive structure, *m*_*1*_ and *m*_*2*_ are the equivalent masses of the two vibrating parts, and *c*_*1*_ and *c*_*2*_ are the damping ratios of the two vibrating parts.

Solving the above system of equations yields the gain (*Gain*_*s*_) and phase shift (${\varphi }_{s}$) of the sensitive units (the rigid magnet) with respect to the input of the sensor system.

6 $$|Gai{n}_{s}(j\omega )|=\frac{\sqrt{1+4{\beta }^{2}{\chi }^{2}}}{\sqrt{{[(1-{\alpha }^{2})(1-{\chi }^{2})-\delta {\chi }^{2}]}^{2}+4{\beta }^{2}{\chi }^{2}(1-{\delta }^{2})}}$$

7 $${\varphi }_{s}(j\omega )=\arctan \frac{(1-{\alpha }^{2})(1-{\chi }^{2})-\delta {\chi }^{2}+4{\beta }^{2}{\chi }^{2}(1-{\alpha }^{2})}{2\beta \chi \{[(1-{\alpha }^{2})(1-{\chi }^{2})-\delta {\chi }^{2}]-(1-{\alpha }^{2})\}}$$

where

$$\alpha =\frac{{\omega }^{2}{m}_{2}}{{k}_{2}},\;\beta =\frac{{c}_{1}}{2{m}_{1}\sqrt{{k}_{1}/{m}_{1}}},\;\chi =\frac{{\omega }^{2}}{\sqrt{{k}_{1}/{m}_{1}}},{\rm{and}}\,\delta =\frac{{m}_{2}}{{m}_{1}}$$

Optimization of the above parameters yields the modal frequencies of each order of the sensor and ensures that the dynamic range of the sensor covers the signal bandwidth.

To simplify the movement of the magnet, we assume that it moves with a constant acceleration and that the speed of the magnet can be determined by Eq. (8).

8 $$v=\sqrt{{v}_{0}^{2}+2a(z-{z}_{0})}$$

where *z*_0_, *v*_0_ and *a* are the initial position of the magnet, the initial velocity of the magnet, and the acceleration of the magnet, respectively.

According to Faraday’s law of electromagnetic induction, the movement of a magnet changes the magnetic field of the coil, allowing mechanical energy to be converted into electrical energy. The complete operating mechanism of the FWMFS in one cycle is described in Fig. 2c. This process involves four main steps. In the initial position shown in Fig. 2c (i), the magnet remains stationary and suspended under the constraints of the soft adhesive layer and soft top layer, and the magnetic flux in the flexible coils does not change; thus, no current is generated. When the FWMFS is subjected to an external mechanical load in the longitudinal direction, the magnet starts to move closer to the coils in response to the external mechanical pressure change, and the magnetic flux distribution through the coils changes, causing current to flow through the coils, as shown in Fig. 2c (ii). This process continues until the magnet moves to the bottom position in Fig. 2c (iii), at which point the magnet and the coils remain relatively stationary to each other, stopping the current. When the external mechanical load is released, the magnet moves upward to the state shown in Fig. 2c (iv) due to the recovery force of the soft top layer. As the direction of magnetic flux change is opposite to the state in Fig. 2c (ii), the current through the coils is also opposite. The magnet moves to the top position and stops returning to the state in Fig. 2c (i), and no current is generated in the coils.

Figure 2b shows that multidimensional sensing of pressure sensors can be realized by controlling the adjustable magnet arrangement. The magnetic field distributions of the longitudinal and transverse magnets are symmetrical. The spatial distribution of the magnetic field changes, causing instantaneous changes in the local magnetic field strength $(\varPhi B=\oint \overrightarrow{B}\cdot d\overrightarrow{S})$ above the coil when the sensor is subjected to an external load force. Here, *B* is the magnetic field strength, and *S* is the effective area of the coils. Based on Faraday’s Law, the induced voltage is $V=-\frac{d\varPhi B}{dt}$.

For sensors with transverse magnets, as defined in this paper, if the north pole of the sensor magnet is forced in the x direction, the value of $\varDelta \varPhi B$ is negative, and a positive voltage is generated in the coil when the external force is unloaded correspondingly. For sensors with magnets arranged longitudinally, this law still applies in the z-direction.

The coil and magnet are the key factors affecting the performance of the sensor. The outputs of the magnet and coils are analyzed theoretically and simulated numerically to obtain the optimal FWMFS parameters. A simplified model of the FWMFS is shown in Fig. S1. The magnetic field source of the FWMFS is a cylindrical Nd_2_Fe_14_B permanent magnet with a diameter of 2.5 mm and a height of 4 mm, Br = 1.46. A three-dimensional polar coordinate system from the top magnet is used to analyze the strength of the magnetic field around the magnet. The expressions for the x, y, and z components of the magnetic field intensity at any point of M (r_0_, θ_0_, z_0_) of the magnetic field, derived based on the basis of the equivalent current model, are expressed by Eq. (9).

9 $$\begin{array}{c}{B}_{x}=\frac{{B}_{r}}{4\pi }\mathop{\displaystyle\int }\limits_{0}^{h}\mathop{\displaystyle\int }\limits_{0}^{2\pi }\frac{{r}_{0}(z-{z}_{0})\cos \theta }{K}d\theta d{z}_{0}\\ {B}_{y}=\frac{{B}_{r}}{4\pi }\mathop{\displaystyle\int }\limits_{0}^{h}\mathop{\displaystyle\int }\limits_{0}^{2\pi }\frac{{r}_{0}(z-{z}_{0})\sin \theta }{K}d\theta d{z}_{0}\\ {B}_{z}=\frac{{B}_{r}}{4\pi }\left\{\begin{array}{c}\mathop{\displaystyle\int }\limits_{0}^{h}\mathop{\displaystyle\int }\limits_{0}^{2\pi }\frac{-{r}_{0}(x-{r}_{0}\,\cos \theta )\cos \theta }{K}d\theta d{z}_{0}\\ -\mathop{\displaystyle\int }\limits_{0}^{h}\mathop{\displaystyle\int }\limits_{0}^{2\pi }\frac{{r}_{0}(y-{r}_{0}\,\sin \theta )\sin \theta }{K}d\theta d{z}_{0}\end{array}\right\}\end{array}$$

$K={[{(x-{r}_{0}\cos \theta )}^{2}+{(y-{r}_{0}\sin \theta )}^{2}+{(z-{z}_{0})}^{2}]}^{3/2}$, *B*_*x*_, *B*_*y*_, and *B*_*z*_ are the components of the magnetic induction intensity $\overrightarrow{B}$ at *x*, *y*, and *z*, respectively; *B*_r_ is the magnet remanence; *z* is the magnet displacement; and *h* is the thickness of the magnet.

The voltage induced by the changing magnetic field in flexible stacked coils can be mathematically expressed by Eq. (10).

10 $${V}_{oc}=-N\frac{d(\int \overrightarrow{B}\cdot \overrightarrow{d}S)}{dt}=-NS\frac{dB}{dz}\cdot \frac{dz}{dt}=-NS\frac{dB}{dz}v$$

where *N* is the number of coil turns.

The magnetic field distribution on the bottom surface of a cylindrical magnet with a radius of 1.25 mm and a height of 4 mm is shown in Fig. 2d. The magnetic field density (MFD) and the rate of change of the magnetic field (dB/dz) in the axial direction are depicted in Fig. 2e at distances ranging from 0 mm to 6 mm below the magnet. The results indicate a significant magnetic field strength and a considerable rate of change in the magnetic field in the 0 to 2 mm range. Therefore, the coils are constrained to move closer to the magnet surface. Assuming that the magnet performs an up-and-down reciprocating sinusoidal motion around the equilibrium position, a numerical simulation of the output of the coil is performed with a vibration acceleration of 9.8 m/s^2^ and an amplitude of 2 mm. The effects of the magnet height and radius on the voltage of the FWMFS are shown in Fig. 2f, g. The results show that as the height of the magnet increases, the peak voltage increases, and as the radius of the magnet increases, the peak voltage first increases and then decreases. When the radius is 1.25 mm and the height is 4 mm, the output voltage of the FWMFS is maximized. Therefore, in the simulation of the coil parameters, the parameters of the magnet are set to a radius of 1.25 mm and a height of 4 mm. The number of coils also affects the voltage of the FWMFS. Figure 2h reveals the relationship between the different positions of the double-layer coils and the performance of the FWMFS. The results show that the output of the double-layer coils gradually decreases as the coil distance, defined as *p*, increases due to the significant attenuation of the magnetic field. When *p* is 3 mm, the output of the coil tends to zero. As shown in Fig. 2i, the output voltage of the FWMFS can be greatly improved by increasing the number of layers of the coil, but this approach has limitations. When the number of coil layers is 10, the output voltage reaches a maximum of ~3 mV.

**Fig. 3:**
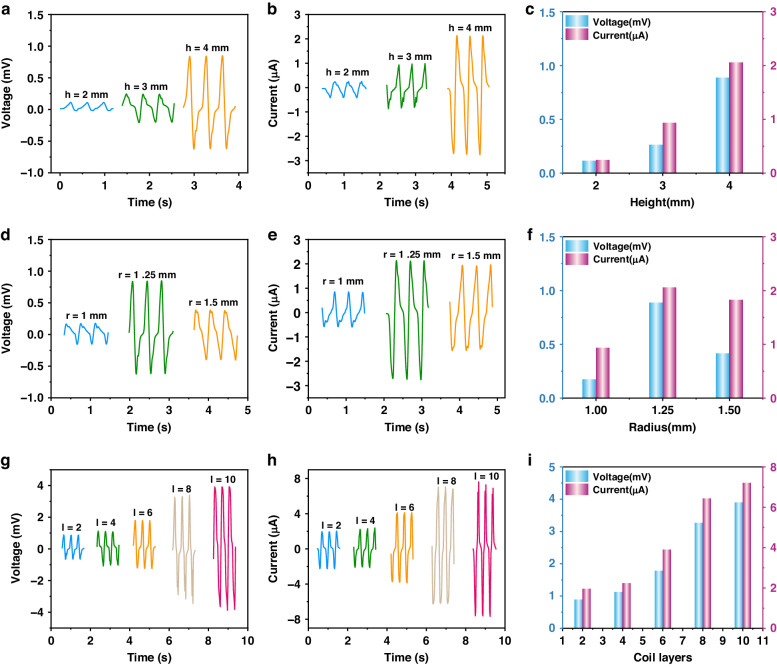
Optimization of the FWMFS sensing performance. Voltage (**a**) and current (**b**) of the FWMFS with different magnet heights. **c** Influence of the magnet height on the output performance. Voltage (**d**) and current (**e**) of FWMFS with different magnet radii. **f** Influence of the magnet radius on the output performance. Voltage (**g**) and current (**h**) of the FWMFS with different flexible coil layers. **i** Influence of the flexible coil layers on the output performance

Based on the above analysis, Fig. 3 shows the FWMFS performance under the different parameters. Fig. 3a, c reveal the relationship between the magnet height and the FWMFS output voltage and current. The results show that the output is greatest when the length of the magnet column is 4 mm. Fig. 3d, f show the relationships between the magnet radius and the FWMFS output voltage and current. The results show that the optimal radius of the magnet is 1.25 mm. Fig. 3g, i demonstrate that the output voltage and current of the FWMFS reach maxima of 4.1 mV and 7.2 µA, respectively, when the number of coil layers is 10. These results are consistent with the simulated analysis.

### Performance of the FWMFS

**Fig. 4:**
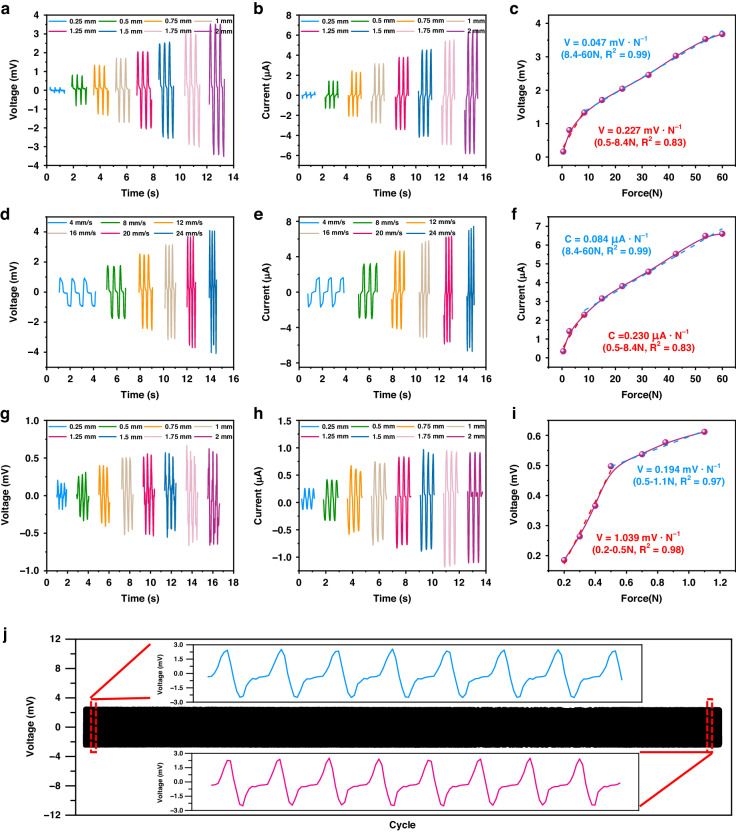
Characterization of the FWMFS when the FWMFS magnet is arranged longitudinally. Voltage (**a**) and current (**b**) of the FWMFS at different compression distances. **c** Voltage response curve of the FWMFS to the pressure inputs. Voltage (**d**) and current (**e**) of the FWMFS at different pressing speeds. **f** Current response curve of the FWMFS to pressure inputs. When the FWMFS magnet is arranged transversely. Voltage (**g**) and current (**h**) of the FWMFS at different compression distances. **i** Voltage response curve of the FWMFS to the pressure inputs. **j** Voltage cycling stability

Figure 4 shows the output performance of the FWMFS with different magnet arrangements. In Fig. 4a, b, the voltage and current of the FWMFS were tested at different compression distances when the magnet was arranged longitudinally. The results show that the voltage and current increase linearly with increasing compression distance, the voltage increases from 0.2 mV to 3.8 mV, and the current increases from 0.4 μA to 6 μA. This is because as the compression distance increases, the magnet approaches the coil, and the magnetic flux through the coil increases, increasing the output. The output of the FWMFS based on the principle of electromagnetic induction is also strongly affected by the compression speed. Figure 4d, e illustrates the voltage and the current as functions of compression speed at a compression distance of 2 mm. The results show that the voltage and the current increase linearly with compression speed, and the maximum values are 4.1 mV and 7.2 µA, respectively, at a compression speed of 24 mm/s. As shown in Fig. 4c, f, the FWMFS exhibited high voltage sensitivities of 0.227 mV/N (0.5–8.4 N) and 0.047 mV/N (8.4–60 N) in response to normal forces in the range of 0.5 N to 60 N. The current sensitivities are 0.230 μA/N (0.5–8.4 N) and 0.084 μA/N (8.4–60 N). When the FWMFS magnet is arranged transversely, the voltage and current of the FWMFS reach a maximum at a compression distance of 2 mm, which are 0.5 mV and 1.0 μA, respectively (Fig. 4g, h). The maximum voltage and current are 0.6 mV and 1.0 μA, respectively, at a pressing speed of 12 mm/s, as shown in Fig. S2a, b. As shown in Fig. 4i and Fig. S2c, the lateral force of the FWMFS ranges from 0.2–1.1 N. The voltage sensitivities are 1.039 mV/N (0.2–0.5 N) and 0.194 mV/N (0.5–1.1 N), and the current sensitivities are 1.560 μA/N (0.2–0.5 N) and 0.339 μA/N (0.5–1.1 N). The cycle performance of the FWMFS was tested at a compression distance of 1.5 mm and a force of 33.2 N. The voltage and current show no significant changes after 5000 cycles, demonstrating perfect stability, as shown in Fig. 4j and Fig. S2d.

### Applications in normal force sensing

**Fig. 5:**
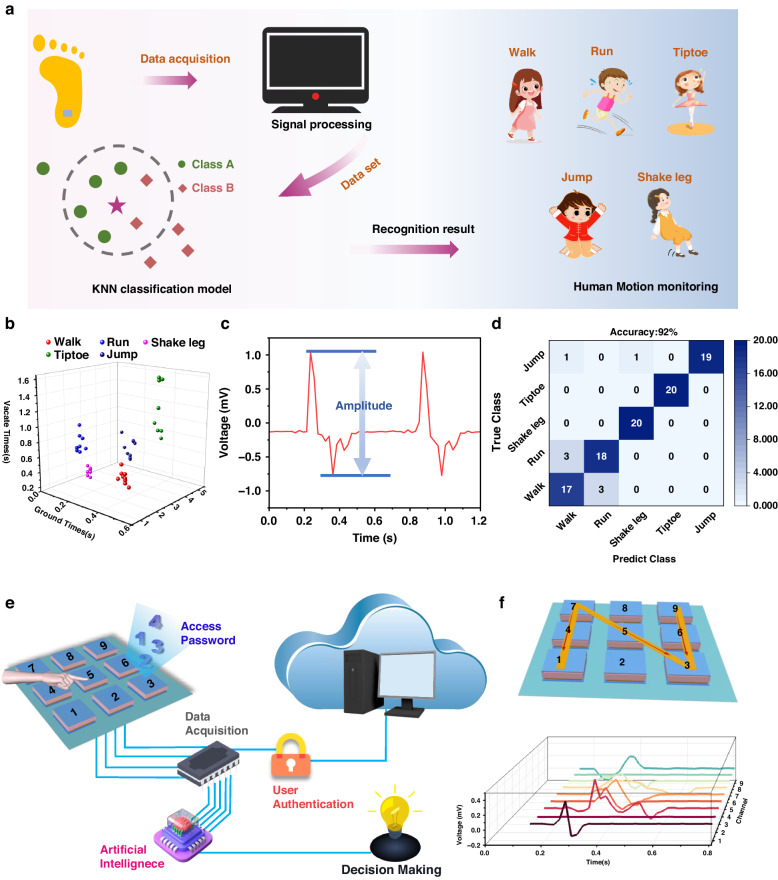
Normal force sensing applications. **a** Mechanism of FWMFS using k-NN classification for motion recognition. **b** Schematic of vacation/groundtime for five different human movements. **c** Feature extraction of the voltage signal using the find-peak method. **d** Confusion matrix of the human movement recognition results. **e** Concept of an artificial intelligence biometric authentication system. **f** Writing the letter “N” above the array

The FWMFS has potential applications in human finger and plantar pressure monitoring due to its wide range. The FWMFS was attached to the left button of a mouse to monitor different levels of finger pressure, such as heavy tapping, medium tapping, light tapping, and sliding, as shown in Fig. S3. Furthermore, FWMFSs are used as plantar pressure sensors to comprehensively monitor human movement postures, combining feature extraction and machine learning methods to classify typical movements. As shown in Fig. 5a, the FWMFS is placed on the sole of a shoe to collect the pressure signal, which is used as a source for machine learning data analysis. The five typical movement states of humans include walking, running, leg shaking, tiptoeing and jumping, each of which has specific electrical signal waveforms, as shown in Fig. S4. Behavior analysis can be performed by measuring the peak amplitude of the signals, the frequency and the time intervals. Figure 5b shows the motion time, recovery time and motion intensity of the collected signal, and the data are available in Table 1 (Supplementary information). Machine learning based on the subspace k-nearest neighbors (k-NN) algorithm is introduced into the human motion monitoring system to realize human motion state analysis and accurate classification. Cross-validation is employed for selecting the best value of k. The k-NN machine learning model has two parts: model training and model testing, that is, supervised learning and real-time posture recognition. Feature values are attributes or characteristics of independent observable objects and need to be extracted before the algorithm is constructed. Selecting feature values is key to the effectiveness of machine learning algorithms. The peaks and troughs of the signal are typical, effective and easily acquired data features for linear classification. Figure 5c shows the peaks and troughs of the running voltage as feature values for human motion recognition. Here, the value of k is 3. A total of 200 samples were used for training and classification, and the scatter plot and confusion matrix plot are plotted in Fig. S5 and Fig. 5d, respectively. The results show that the accuracy rate for human motion recognition is 92%. A 3*3 array keyboard was created to convert the mechanical energy of typing into electrical signals that are fed into the AI system, as shown in Fig. 5e. Biometrics and authentication are based on an individual’s typing rhythm behavior to prevent password vulnerability. Additionally, the array keyboard can be used for trajectory recognition; when the letters “N”, “I”, “Z” and “U” are written above the array, the output response of each sensor is shown in Fig. 5f and Fig. S6.

### Applications in multidimensional force sensing

**Fig. 6:**
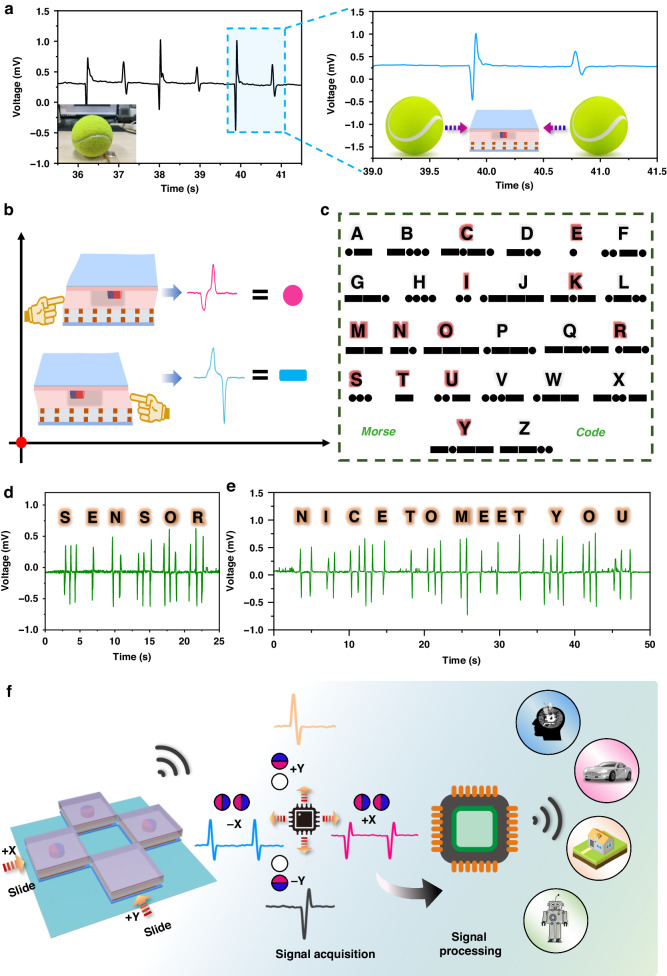
Applications in multidimensional force sensing. **a** Signals of the tennis ball rolls in different directions. **b** Principle of Morse Code communication. **c** International Morse Code table. The electrical signals of the Morse Code ‘Sensor’ (**d**) and ‘Nice to meet you’ (**e**). **f** Schematic of the FWMFS array used to sense the HMI interface in four directions

The multidimensional force sensing performance of the FWMFS has great potential for monitoring the direction of motion of tiny objects. For example, when the FWMFS is fixed on the ground and a tennis ball is rolled from different directions, the signals of “+/−“ and “−/+” can accurately respond to changes in the direction and intensity of the tennis ball motion, as shown in Fig. 6a. Directional awareness of signals can be tailored to optimize communication systems such as Morse Code, as shown in Fig. 6b. According to International Morse Code (Fig. 6c), controlling the tap direction produces points and lines that can be identified as Morse Code, using “sensor” and “nice to meet you” as examples in Fig. 6d, e, respectively. A multidimensional directional sensing system is formed by exploiting the two-dimensional directional sensing capability of the FWMFS. FWMFSs are arranged in an array, as shown in Fig. 6f, which can distinguish the input from the four directions simultaneously. This system is promising for human‒machine communication and intelligent perception.

## Conclusions

In summary, inspired by bones embedded in the muscle structure of human fingers, a flexible wide-range multidimensional force sensor with multilayer stacked MEMS coils based on electromagnetic induction is proposed. The adjustable position of the magnetic column allows force detection in different directions. Various parameters of rigid magnets and flexible coils are compared to optimize the FWMFS configuration, and the corresponding sensing mechanism is analyzed via numerical simulation. The simulation results show that the FWMFS output performance is best when the magnet radius is 1.25 mm, the height is 4 mm and the number of coil layers is 10. The output voltage and current of the FWMFS reached a maximum of 4.1 mV and 7.2 µA, respectively, after the experiment, effectively resolving the trade-off between miniaturization and output capacity in flexible electromagnetic sensors. The FWMFS has longitudinal voltage sensitivities of 0.227 mV/N (0.5–8.4 N) and 0.047 mV/N (8.4–60 N) with a wide normal force measurement range up to 60 N and lateral sensitivities of 1.039 mV/N (0.2–0.5 N) and 0.194 mV/N (0.5–1.1 N) with a detection range up to 1.1 N. The FWMFS can be widely applied to single normal force sensing and multidimensional force sensing. Moreover, owing to its wide range, it can not only be attached to a mouse to monitor different levels of pressure in the fingers but also be integrated into the sole of a shoe to measure plantar pressure. Combined with machine learning algorithms (k-NNs), the FWMFS was used to collect and analyze five plantar pressure signals from human motion, achieving an accuracy rate of 92%. A 3*3 array has been developed and integrated with a data acquisition and signal processing system for user identification and authentication combined with an artificial intelligence algorithm. Furthermore, the FWMFS array can be used for self-powered 3D trajectory sensing. We also demonstrated the application of the proposed sensors in monitoring the direction of ball movement, Morse Code, and artificial intelligence.

## Materials and methods

### Fabrication of flexible multilayer stacked coils

Double-layer flexible coils were fabricated, and then the multilayer coils were stacked. Double-layer coils (0.035 mm in line width, 0.035 mm in line space) were fabricated using flexible circuit board technology (Shenzhen Zhongsheng Electronics Co., Ltd.) in seven steps (Supplementary Information Fig. S7 and Note 1). The multilayer coils were then sequentially bonded using high-temperature adhesive.

### Fabrication of the soft adhesive layer, rigid magnet and support layer

The 15 × 15 mm square templates with different thicknesses were fabricated by a 3D printer (ZRapid Tech., Ltd.). A mixture of Sylgard 184 silicone elastomer base and curing agent in a weight ratio of 10:1 (Dow Corning Co., Ltd.) was defoamed under vacuum for 20 min at room temperature before being poured into the mold. The Nd_2_Fe_14_B magnet was placed onto the PDMS and then cured at 80 °C for 2 h. The cured PDMS with a rigid magnet was peeled off from the mold. The cured PDMS without a rigid magnet was cut into a 15 × 15 mm square film with a 5 mm radius central hole, which was used as the support layer.

### Fabrication of the FWMFS

Liquid-state PDMS was coated on the upper and lower sides of the support layer to bond the soft adhesive layer and the stacked flexible coils, respectively. The soft top and bottom layers are polyether-based TPU films (BASF, China) used to encapsulate the entire structure.

### Characterization

SEM images of the flexible coils were obtained by field-emission scanning electron microscopy (FE-SEM, Hitachi SU8020, Japan). The surface elemental compositions and distributions were measured using an energy-dispersive X-ray spectroscopy (EDX) detector attached to a scanning electron microscope, as shown in Fig. S8.

### Measurement

The performance of the FWMFS was tested using a measurement setup based on a servo motor, as shown in Supplementary Information Fig. S9. The system can be programmed in a control box to move the compression blocks at different compression speeds and compression distances by converting the rotary motion of the servo motor into linear motion. The FWMFS was mounted at the end of the base, and the output signal was collected by a Keithley 2611B Source Meter and LabView software. A commercial pressure sensor (HZC-T-300N, Bengbu Chengying Sensor Co., Ltd., China) was mounted on the compression block, and the pressure data were collected by the upper computer software during movement.

### Supplementary information

Supplementary information
