# Supplementary material for: Flexible wide-range multidimensional force sensors inspired by bones embedded in muscle
Source: Microsyst Nanoeng. 2024 May 22;10:64. doi: 10.1038/s41378-024-00711-7 (PMC11111798; doi:10.1038/s41378-024-00711-7)
Supplement: Supplementary file 1 — Supplementary information [file 41378_2024_711_MOESM1_ESM.docx]

**Supplement Information**

**Flexible wide-range multidimensional force sensors inspired by bones embedded in muscle**

Jie Zhang^1^, Xiaojuan Hou^1*^, Shuo Qian^2^, Jiabing Huo^1^, Mengjiao Yuan^1^, Zhigang Duan^1^, Xiaoguang Song^1^, Hui Wu^1^, Shuzheng Shi^3,4*^, Wenping Geng^1^, Jiliang Mu^1^, Jian He^1^, Xiujian Chou^1*^

*^1^* *Science and Technology on Electronic Test and Measurement Laboratory, North University of China, Taiyuan 030051, China*

*^2^ School of Software, North University of China, Taiyuan 030051, China*

*^3^ School of Mechanical Engineering, Hebei University of Architecture, Zhangjiakou 075000, China ^4^ HBIS Group Co., Ltd., Shijiazhuang 050023, China*

*Corresponding author

E-mail address: [houxiaojuan@nuc.edu.cn](mailto:houxiaojuan@nuc.edu.cn)


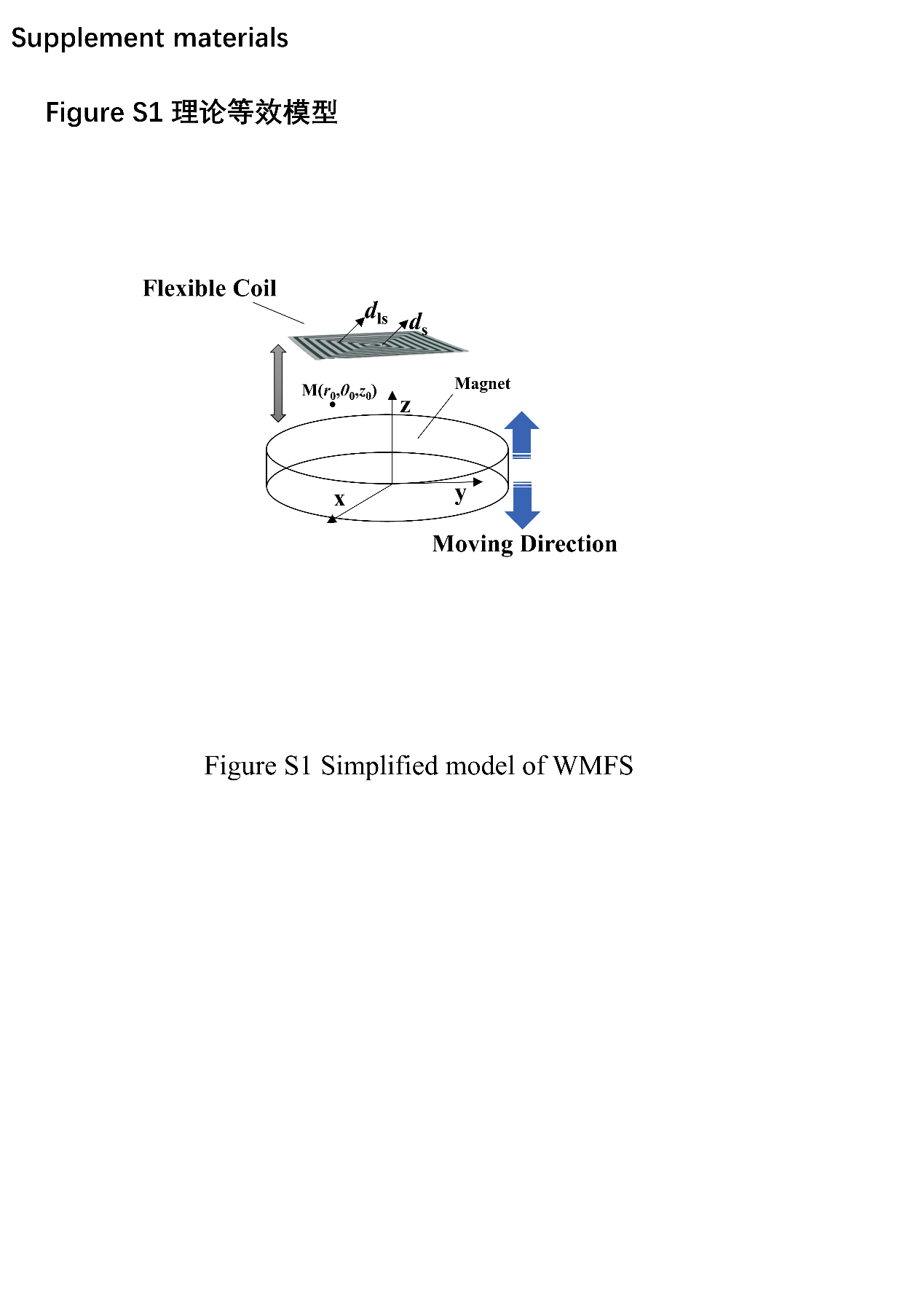


**Figure S1 Simplified model of FWMFS.**


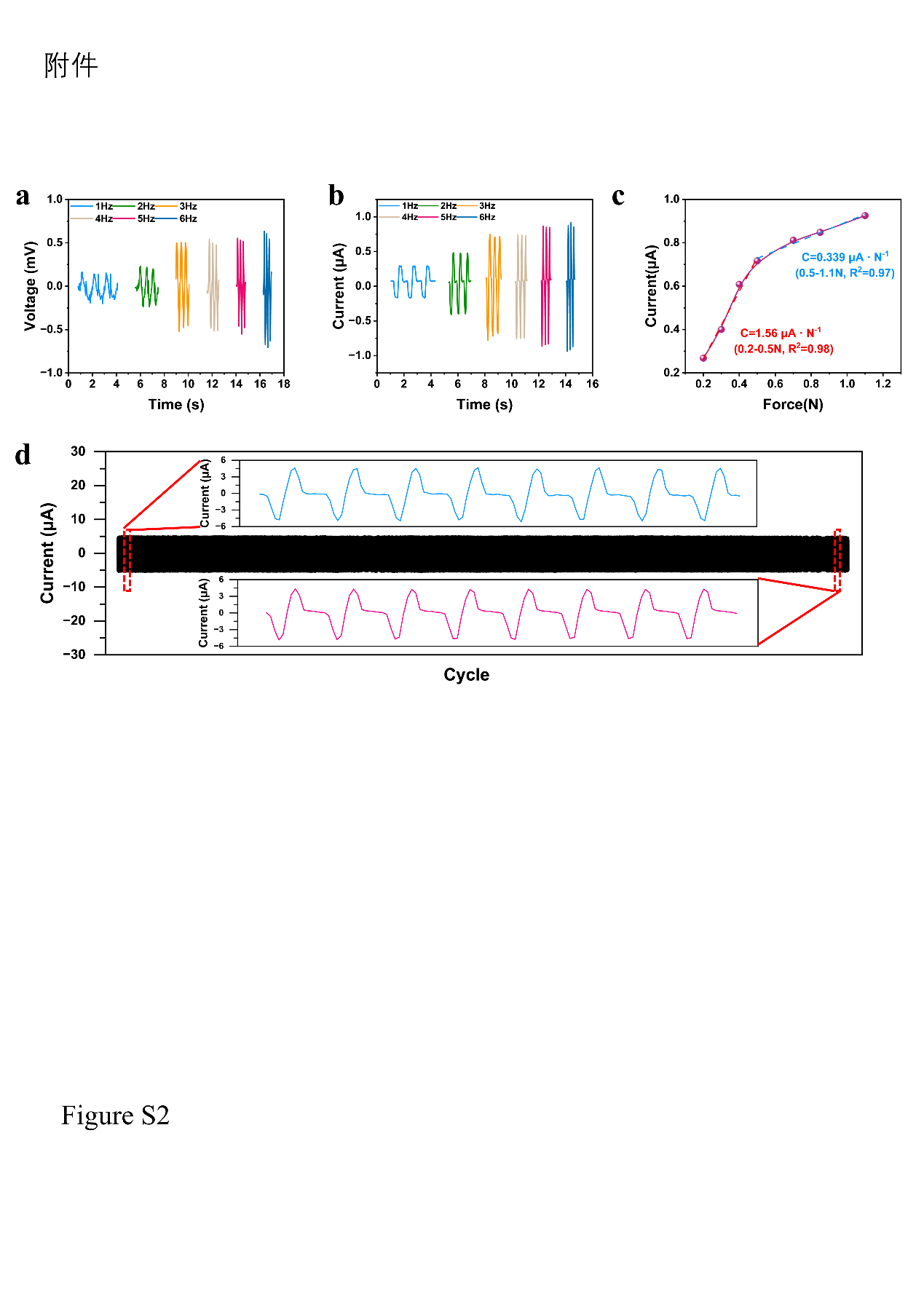


**Figure S2 Characterization of FWMFS.** Voltage(a) and Current (b) of FWMFS with the transverse arrangement of magnets at different frequency. (c) Current sensitivity. (d) Voltage cycling stability.


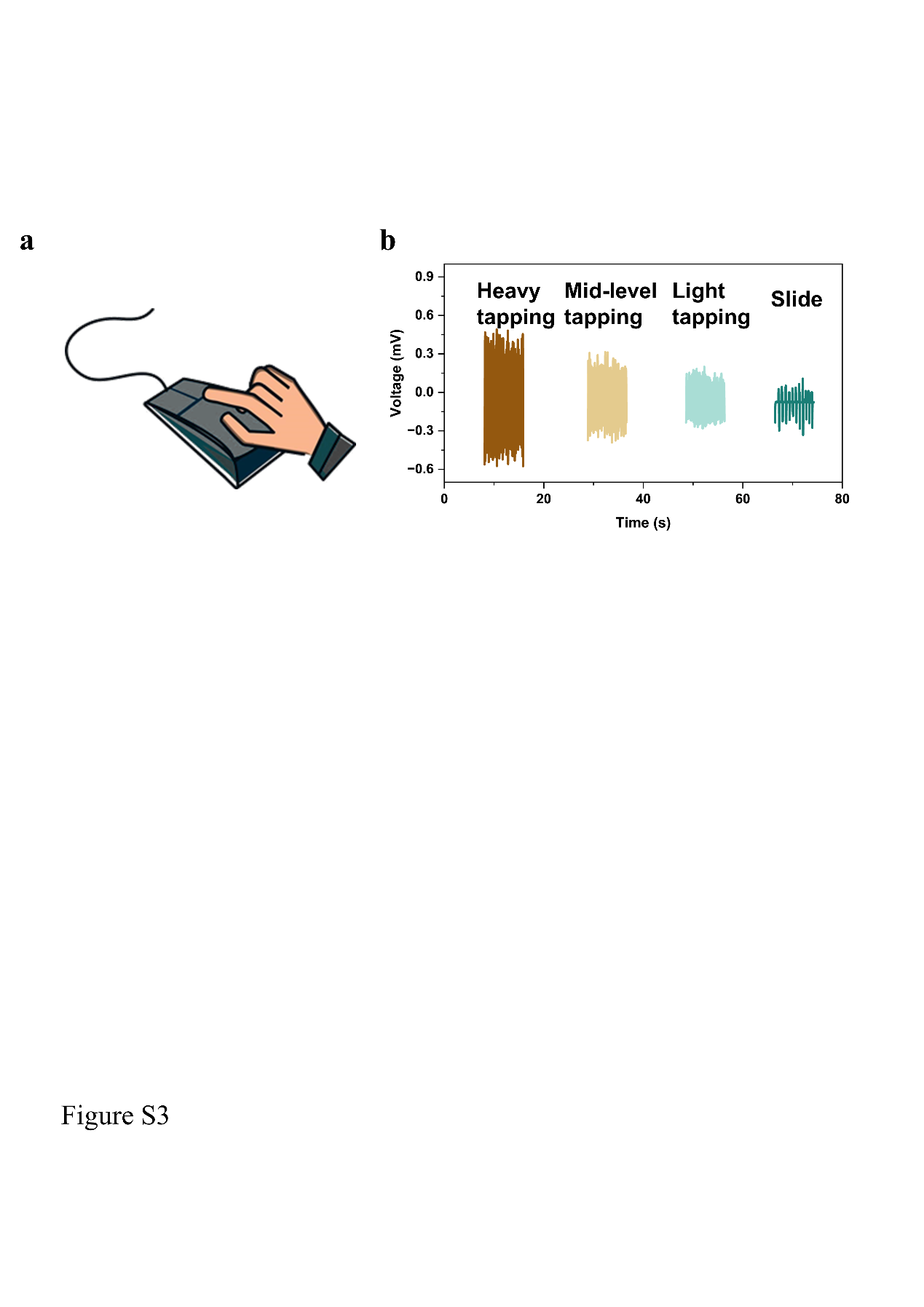


**Figure S3 Output voltage of FWMFS in different mouse pressing modes.**


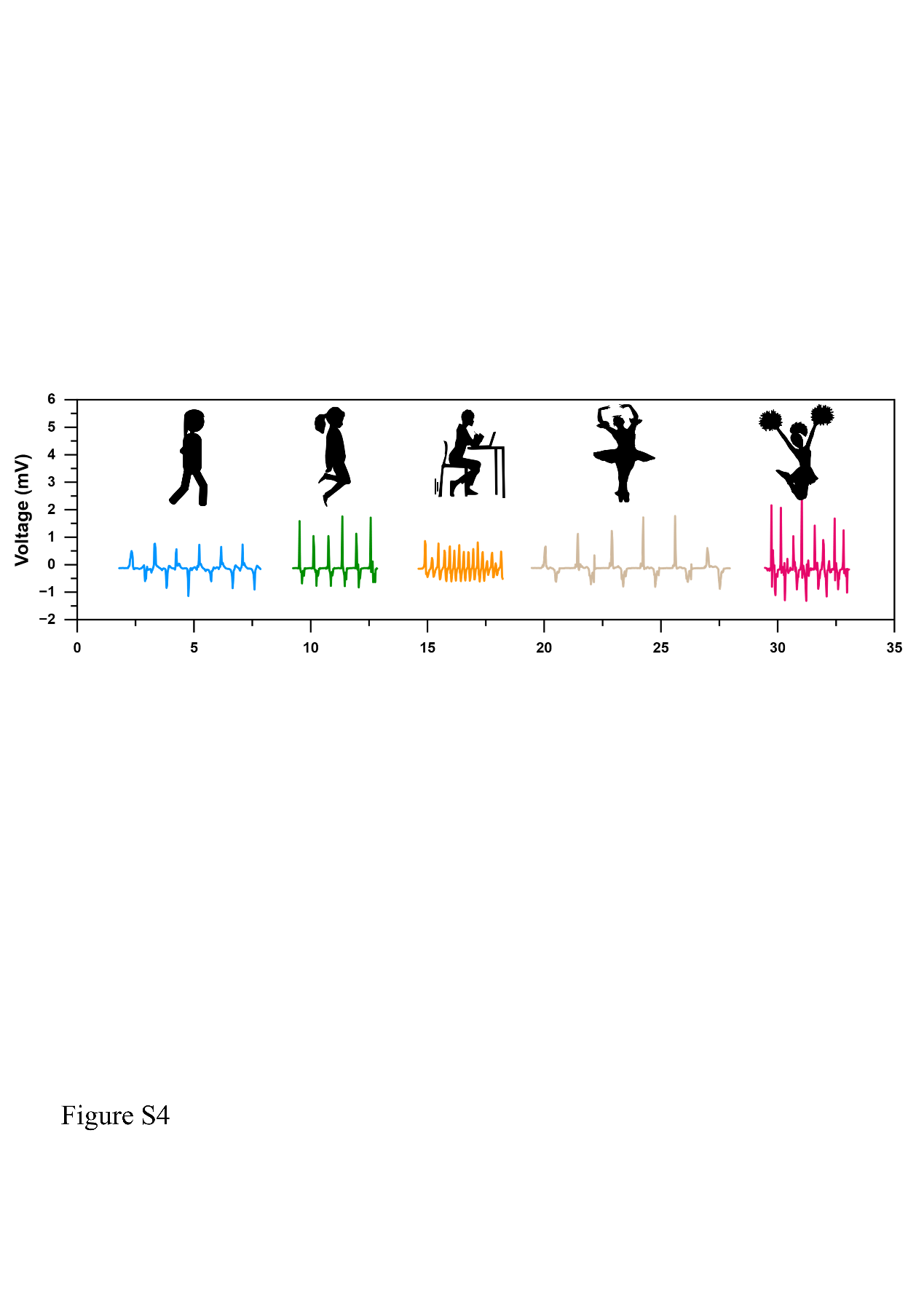


**Figure S4 Output performance of FWMFS with five different human movement**


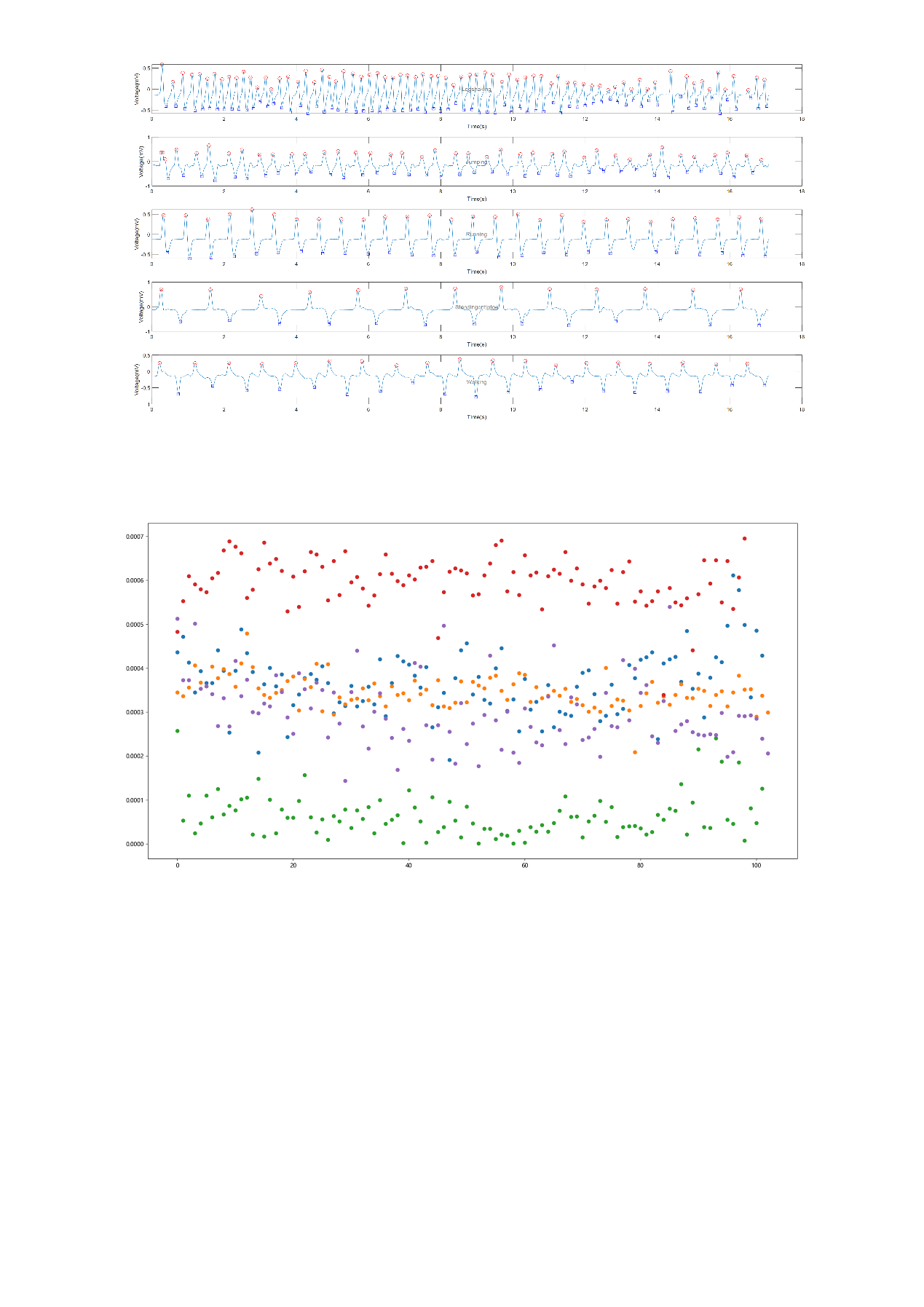


**Figure S5 Scatter plot of human motion recognition results**.


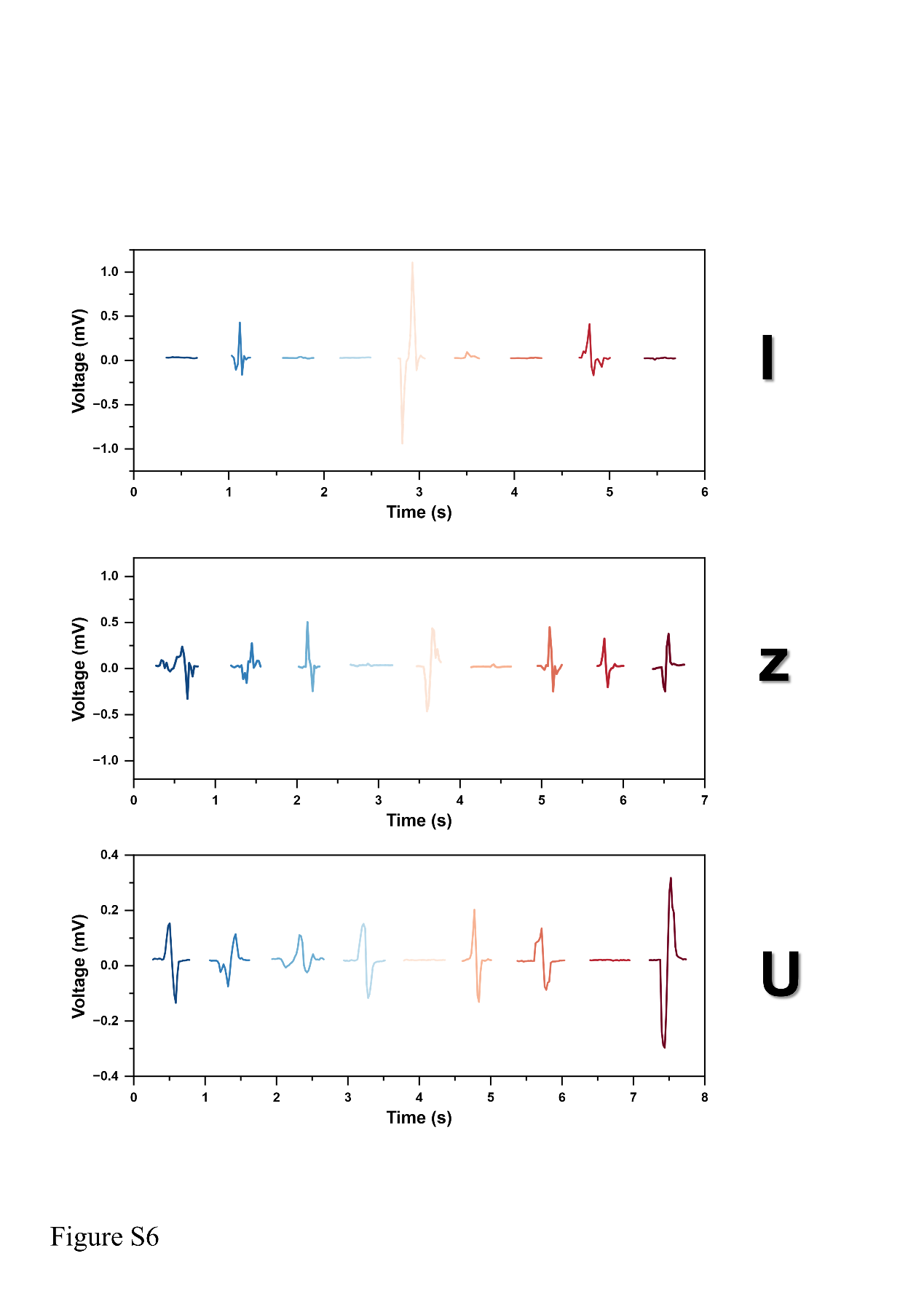


**Figure S6 Writing the letter ‘I’, ‘Z’, ‘U’ above the array.**


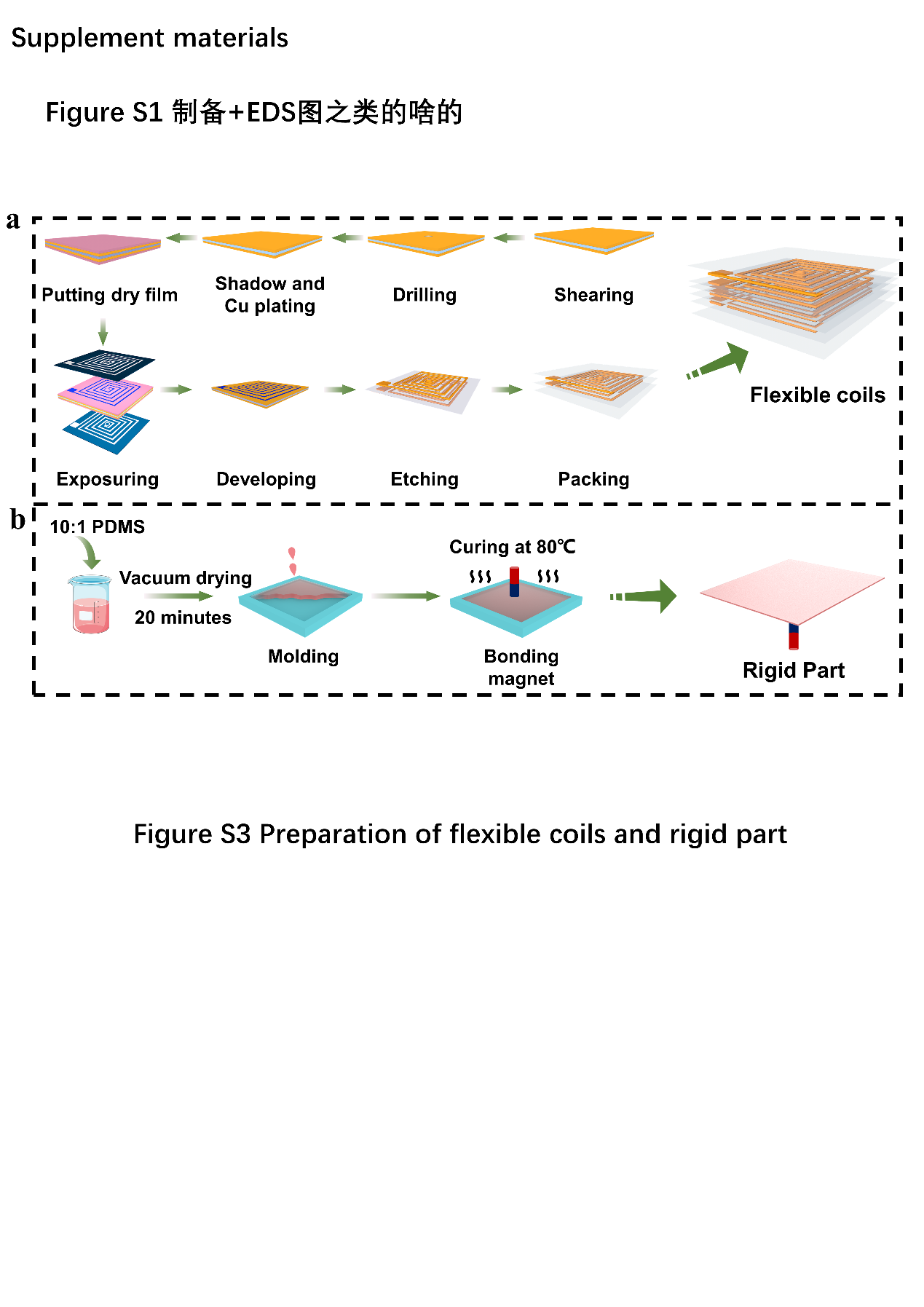


**Figure S7 Fabrication of FWMFS.** The manufacturing process of the multilayer flexible MEMS coil (a) and rigid part(b)

**Note.1** **The manufacturing process of the multilayer flexible MEMS coil.**

1. Shearing. In order to make the printed board as thin as possible, a non-adhesive substrate is used, and the polyimide film is directly combined with the copper foil to make a flexible copper-clad laminate.

2. Drilling. Drilling to leave a center through hole for conduction between the upper and lower layers.

3. Through shadow and cu plating, the upper and lower layers of the coils are interconnected.

4. The dry film is used as an anti-etching medium. After exposure and development, the pattern is transferred and the copper under the unexposed position is protected.

5. A double-layer copper coil is prepared after etching and stripping.

6. The cover layer is composed of polyimide film, adhesive and release paper, which has good flexural properties. It protects flexible circuits from dust, moisture, chemicals, and reduces stress during bending.

7. The assembly of multilayer stacked coils is formed through silver paste conductive adhesive.


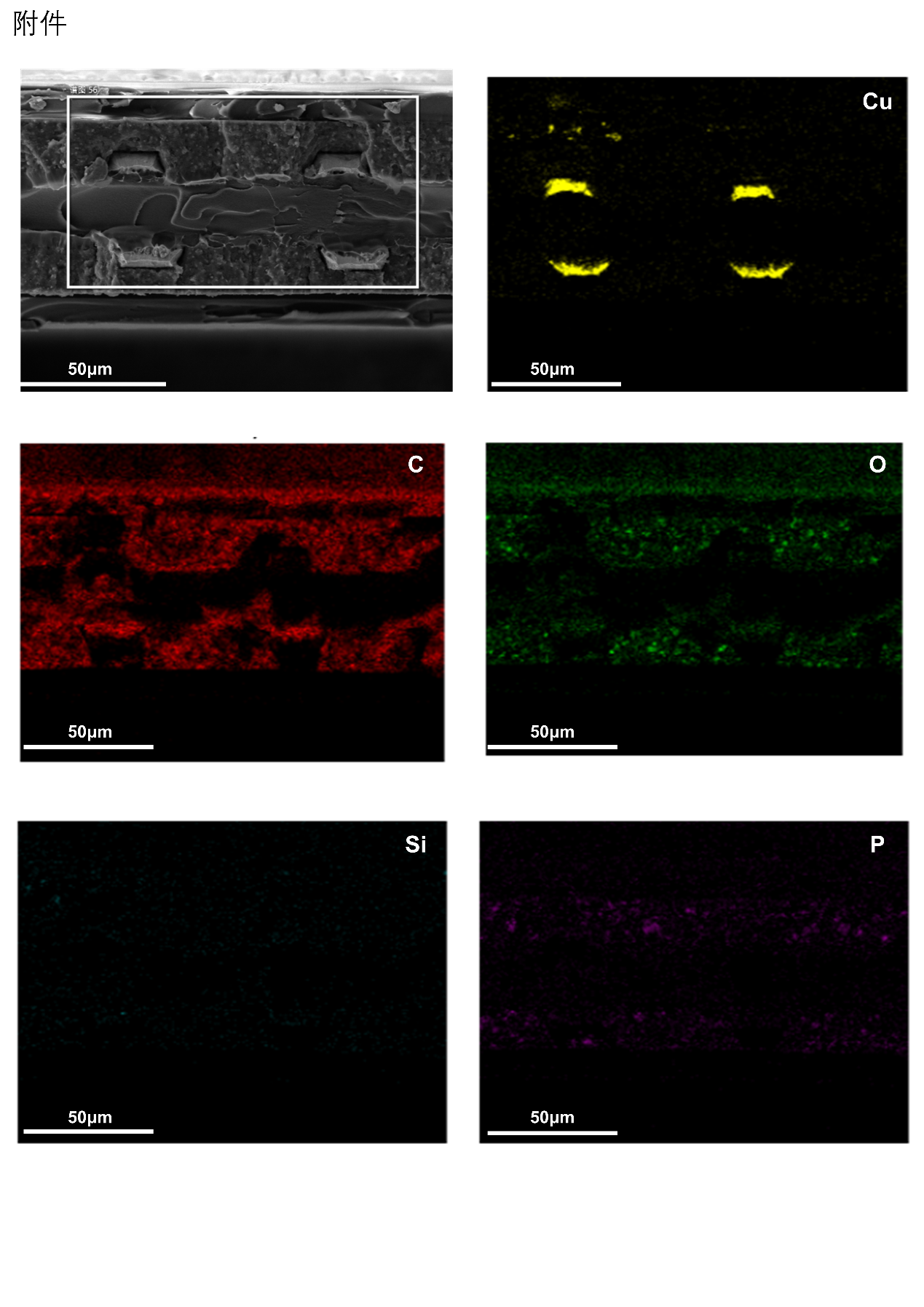


**Figure S8 EDS images of the flexible MEMS coils.**


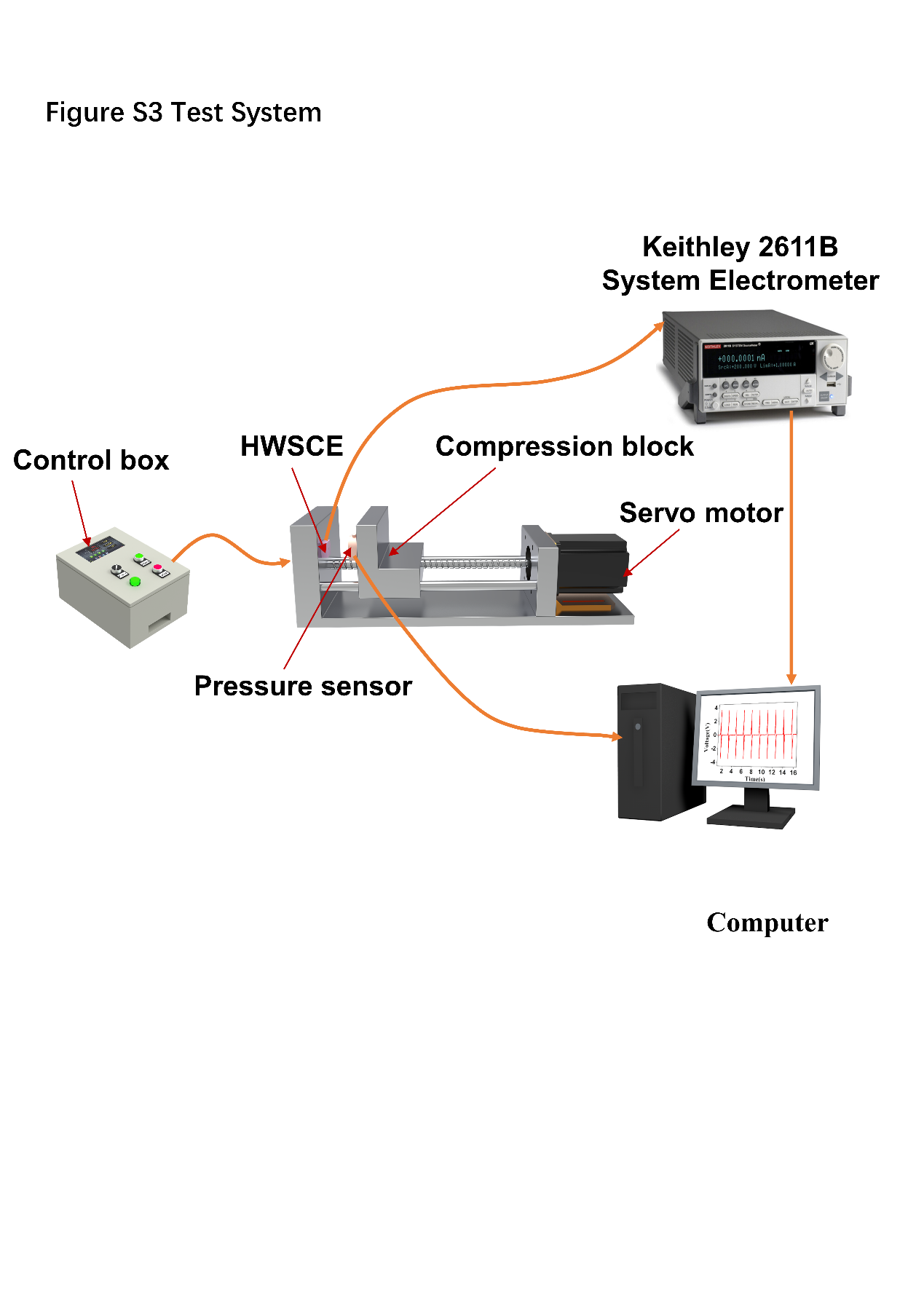


**Figure S9 Schematic of the test process.**

**Table 1 The data of motion time, recovery time and motion intensity.**

| Motion State | Ground Times(s) | Vacate Times(s) |
| --- | --- | --- |
| Walking | 0.499 | 0.394 |
|  | 0.49 | 0.426 |
|  | 0.51 | 0.425 |
|  | 0.469 | 0.425 |
|  | 0.51 | 0.426 |
|  | 0.511 | 0.425 |
|  | 0.533 | 0.425 |
|  | 0.489 | 0.425 |
|  | 0.511 | 0.425 |
| Running | 0.106 | 0.51 |
|  | 0.106 | 0.522 |
|  | 0.085 | 0.531 |
|  | 0.128 | 0.468 |
|  | 0.106 | 0.532 |
|  | 0.084 | 0.489 |
|  | 0.107 | 0.531 |
|  | 0.085 | 0.532 |
|  | 0.149 | 0.468 |
| Leg shaking | 0.064 | 0.149 |
|  | 0.064 | 0.148 |
|  | 0.044 | 0.17 |
|  | 0.064 | 0.148 |
|  | 0.043 | 0.17 |
|  | 0.043 | 0.15 |
|  | 0.064 | 0.17 |
|  | 0.043 | 0.148 |
|  | 0.064 | 0.149 |
| Tipping | 0.425 | 0.83 |
|  | 0.426 | 0.808 |
|  | 0.425 | 0.808 |
|  | 0.404 | 0.851 |
|  | 0.426 | 0.808 |
|  | 0.425 | 0.808 |
|  | 0.447 | 0.787 |
|  | 0.447 | 0.766 |
|  | 0.446 | 0.809 |
| Jumping | 0.17 | 0.341 |
|  | 0.148 | 0.213 |
|  | 0.181 | 0.319 |
|  | 0.149 | 0.298 |
|  | 0.106 | 0.34 |
|  | 0.149 | 0.234 |
|  | 0.17 | 0.34 |
|  | 0.128 | 0.318 |
|  | 0.085 | 0.362 |
